# AI-based automated body composition analysis from whole-body MRI and (PET-) CT: an intermodal comparison from same-day examinations

**DOI:** 10.1186/s40644-026-01134-5

**Published:** 2026-10-24

**Authors:** Julius C. Holzschuh, Lukas T. Rotkopf, Christian Neelsen, Fabian Bauer, Kevin S. Zhang, Ekaterina Menis, Marina Hajiyianni, Elias Karl Mai, Niels Weinhold, Lukas John, Christos Sachpekidis, Antonia Dimitrakopoulou-Strauss, Marc-Steffen Raab, Hartmut Goldschmidt, Johann Jende, Stefan Delorme, Heinz-Peter Schlemmer, Markus Wennmann

**Affiliations:** 1 Department of Radiology, German Cancer Research Centre (DKFZ), Im Neuenheimer Feld 280, Heidelberg, 69120 Germany; 2 Faculty of Medicine and University Hospital Cologne, Institute for Diagnostic and Interventional Radiology, Cologne, Germany; 3 German-Speaking Myeloma Multicenter Group (GMMG), Heidelberg University Hospital, Heidelberg, 69120 Germany; 4 Department of Internal Medicine V, Hematology, Oncology and Rheumatology, Medical Faculty Heidelberg, Heidelberg Myeloma Center, Heidelberg University Hospital, Heidelberg, 69120 Germany; 5 Clinical Cooperation Unit Nuclear Medicine, German Cancer Research Centre (DKFZ), Heidelberg, Germany; 6 Department of Medical Oncology, National Center for Tumor Diseases (NCT), Heidelberg University Hospital, Heidelberg, 69120 Germany

**Keywords:** AI, Deep Learning, PET/CT, MRI, Oncology, Multiple myeloma

## Abstract

**Background:**

To determine intermodal agreement of AI-derived volumetric body composition metrics between [18 F]FDG-PET/CT-derived low-dose CT and whole-body MRI (wb-MRI).

**Methods:**

We retrospectively analyzed same-day whole-body low-dose CT, obtained from [^18^F]FDG-PET/CT, and wb-MRI from consecutive multiple myeloma patients from the prospective GMMG-HD7- and GMMG-HD8-/DSMM-XIX-trials. An automated deep learning pipeline based on the TotalSegmentator model, and its MRI variant were used to segment skeletal muscle (SM), subcutaneous- (SAT) and abdominal visceral adipose tissue (VAT) from CT and MRI, respectively. Volumetric measures and single-slice estimates were derived. Agreement between CT- and MRI-based metrics was assessed using Pearson r, intraclass correlation coefficients (ICC), and Bland-Altman analyses.

**Results:**

Forty-two multiple myeloma patients with same-day PET/CT and wb-MRI were included. For AI-based volumetric body composition metrics, CT and MRI demonstrated high agreement (ICC > 0.95) across all SM, SAT and VAT. However, Bland-Altmann plots revealed systematic modality-related differences between MRI and CT: both SM (bias: -2582 ml, *p* < 0.0001) and VAT (bias: -211 ml, *p* < 0.0001) were significantly lower when extracted from MRI compared to CT, while for SAT, the difference was not statistically significant (bias: -263 ml, *p* = 0.1629). For AI-based single slice analysis, agreement remained high for SAT and VAT (ICC > 0.97), while being lower for SM (ICC 0.87). Bland-Altman plots showed that SM was systematically and significantly lower when obtained from MRI than from CT (bias: -18.4cm^2^, *p* < 0.0001).

**Conclusions:**

Automated body composition measures from (PET-)CT and wb-MRI are highly concordant but show consistent intermodal biases that should be considered when pooling multimodal data or applying modality-agnostic thresholds.

**Clinical trial number:**

Not applicable.

**Supplementary Information:**

The online version contains supplementary material available at https://doi.org/10.1186/s40644-026-01134-5.

## Summary statement

Fully automated deep learning body composition analysis showed strong agreement between same-day low-dose CT from PET/CT and whole-body MRI in newly diagnosed multiple myeloma patients. However, MRI produced systematically lower skeletal muscle and visceral adipose tissue estimates than CT, indicating modality-dependent bias.

**Supplementary Information:**

The online version contains supplementary material available at https://doi.org/10.1186/s40644-026-01134-5.

## Key points

1. Whole-body volumetric quantification of skeletal muscle, subcutaneous adipose tissue, and abdominal visceral adipose tissue can be performed in a fully automated manner from both low-dose CT (from PET/CT) and whole-body MRI acquired on the same day.

2. While correlation was high, tissue-specific, modality-related biases were observed, indicating that values from CT and MRI are not directly interchangeable without accounting for systematic differences in real world clinical practice, particularly for skeletal muscle and visceral adipose tissue.

3. Segmentation quality was predominantly sufficient-to-good on radiologist review with the main error patterns were linked to imaging artifacts and challenging anatomy

**Supplementary Information:**

The online version contains supplementary material available at https://doi.org/10.1186/s40644-026-01134-5.

## Introduction

Alterations in body composition, particularly loss of skeletal muscle and redistribution of adipose tissue, are recognized as clinically important in oncology [1] as these changes have been associated with chemotherapy intolerance [2], increased postoperative complications [3], and reduced survival [4], underscoring their potential role as prognostic biomarkers that extend beyond conventional clinicopathological factors. Accurate and reproducible quantification of body composition may therefore provide a critical adjunct for risk stratification and personalized care in patients with cancer.

Computed tomography (CT), magnetic resonance imaging (MRI), and positron emission tomography-CT (PET-CT) are central to oncologic imaging and offer detailed information on muscle and fat compartments. Traditionally, body composition analyses have relied on single-slice, two-dimensional measurements, most commonly at the third lumbar vertebra, obtained manually or semi-automatically [5, 6]. While these approaches have demonstrated prognostic value, they are limited by slice selection bias, operator dependence, and their inability to capture the complexity of whole-body composition.

The advent of deep learning has transformed the landscape of quantitative imaging. Automated segmentation algorithms now enable rapid, reproducible, and scalable assessment of skeletal muscle and adipose tissue, facilitating volumetric body composition analyses across entire imaging datasets [7, 8]. Such advances raise the prospect of embedding body composition assessment into routine clinical workflows and large-scale research [9, 10]. Yet, despite this progress, a critical knowledge gap remains: the reproducibility of automated body composition measures across imaging modalities.

Previous comparative studies have reported good correlations between CT- and MRI-derived metrics [11, 12, 13]. However, these investigations were largely restricted to single-slice or region-of-interest methods and often relied on manual processing. As a result, the degree to which fully automated, volumetric body composition analyses by state-of-the-art AI-algorithms provide consistent estimates across CT and MRI remains uncertain. Addressing this limitation is essential if imaging-derived body composition measures are to be standardized and adopted as robust biomarkers in oncology.

In this study, we established automated workflows which integrate TotalSegmentator [7] or its MRI variant [8], respectively, to enable the extraction of skeletal muscle and adipose tissue metrics from same-day whole-body CT and MRI examinations. Using this pipeline, we derived both volumetric and single-slice body composition measures and systematically evaluated their agreement, reproducibility, and modality-specific biases.

## Methods

### Study design

**Fig. 1:**
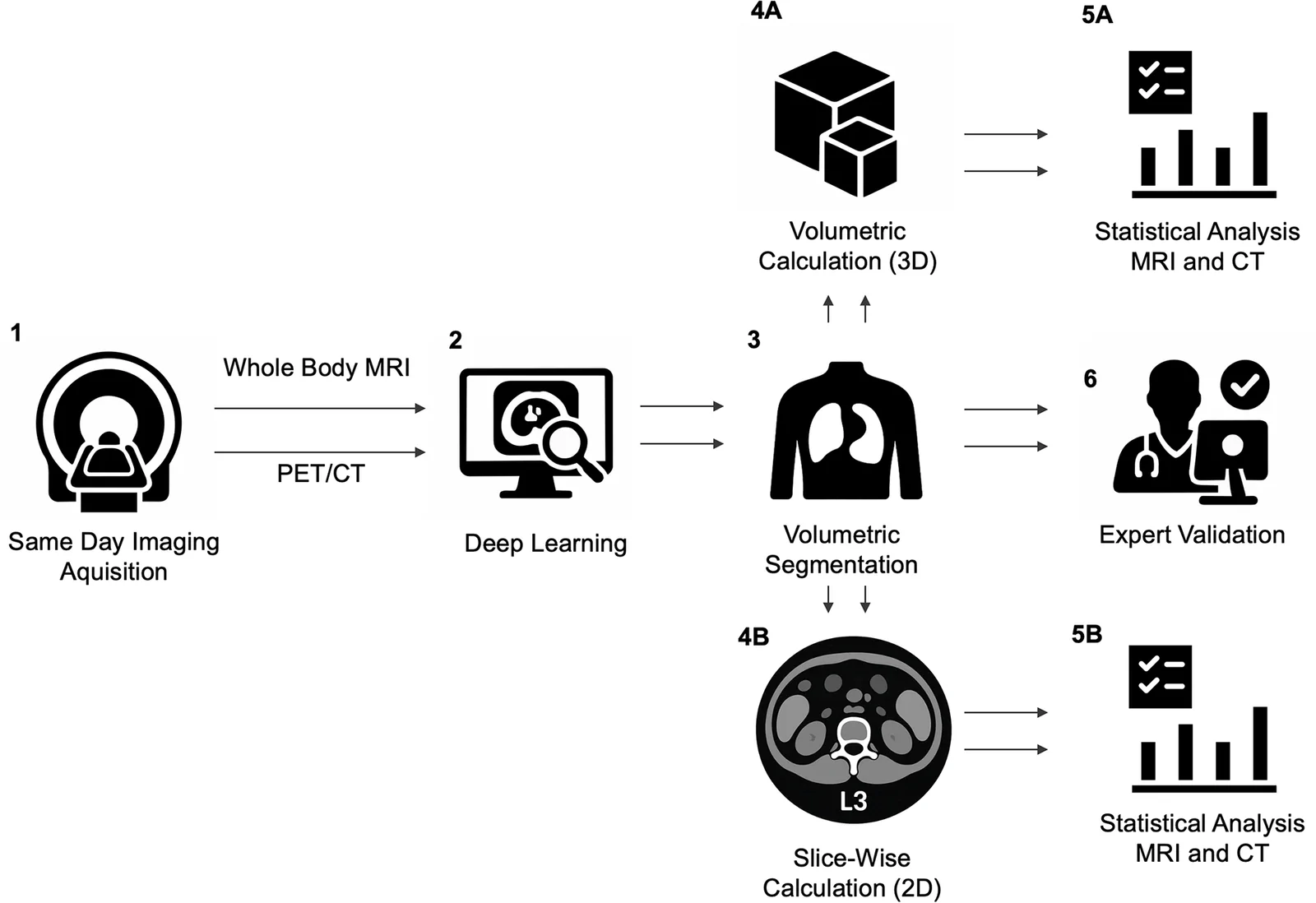
Study design: PET/CT and whole-body MRI were acquired on the same day (1) , and each modality was automatically segmented using deep learning (2), resulting in volumetric segmentations of skeletal muscle, subcutaneous fat, and visceral fat (3). Body composition was quantified by whole-body volumetry (4 A) and slice-based analysis at L3 (4B), with CT and MRI results compared for both approaches (5 A, 5B). Segmentation quality was validated by a board-certified radiologist (6)

A graphical overview over the study is provided in Fig. 1. The study included patients with newly diagnosed multiple myeloma who received same-day PET/CT and whole-body MRI examinations. A total of 123 consecutive patients from the prospective GMMG-HD7- (ClinicalTrials.gov identifier: NCT03617731) [14, 15] and GMMG-HD8-/DSMM-XIX-study (ClinicalTrials.gov identifier: NCT05804032) who had undergone baseline 18 F-FDG-PET-CT at one of two large oncologic imaging centers between 24.10.2018 and 27.02.2025 were screened. Eighty-one patients were ineligible because they had not undergone whole-body MRI at the same day, leaving 42 patients with same day PET-CT and whole-body MRI acquired between March 2019 and February 2025 for inclusion in the analysis (Supplementary Fig. 1). Patient characteristics of the final study cohort obtained from images and scan metadata are summarized in Table 1. This retrospective study was conducted in accordance with the Declaration of Helsinki and approved by the local institutional review board (S-513/2024). All included patients had provided broad consent for the use of their imaging data for research purposes.

### Imaging

Whole-body MRI was performed on a 1.5 T system (Magnetom Aera; Siemens Healthineers, Erlangen, Germany; software version Syngo MR E11) without intravenous contrast. The protocol comprised a coronal T1-weighted turbo spin-echo (TSE) sequence, a coronal T2-weighted short-tau inversion recovery (STIR) TSE sequence, and axial diffusion-weighted echo-planar imaging (DWI). Volumetric body composition analysis was based on the coronal T1-weighted TSE sequence. Whole-body MRI volumes were the composed image generated automatically by the vendor’s software on the scanner console without any additional station-to-station composition performed.

¹⁸F-FDG PET/CT scans were performed on the Biograph System (Siemens Healthineers, Erlangen, Germany), specifically a Biograph 128 Vision Quadra Edge (*n* = 22, 52%), Biograph mCT 40 (*n* = 14, 33%) and Biograph mCT 20 (*n* = 6, 14%). Low-dose CT scans (120 kV, 30 effective mAs) were acquired for both attenuation correction and anatomical co-registration. The CT component with soft tissue kernel reconstruction was used for the CT-based body composition analysis. A detailed description of all technical details of image acquisition can be found in the supplementary.

### Image segmentation

**Fig. 2:**
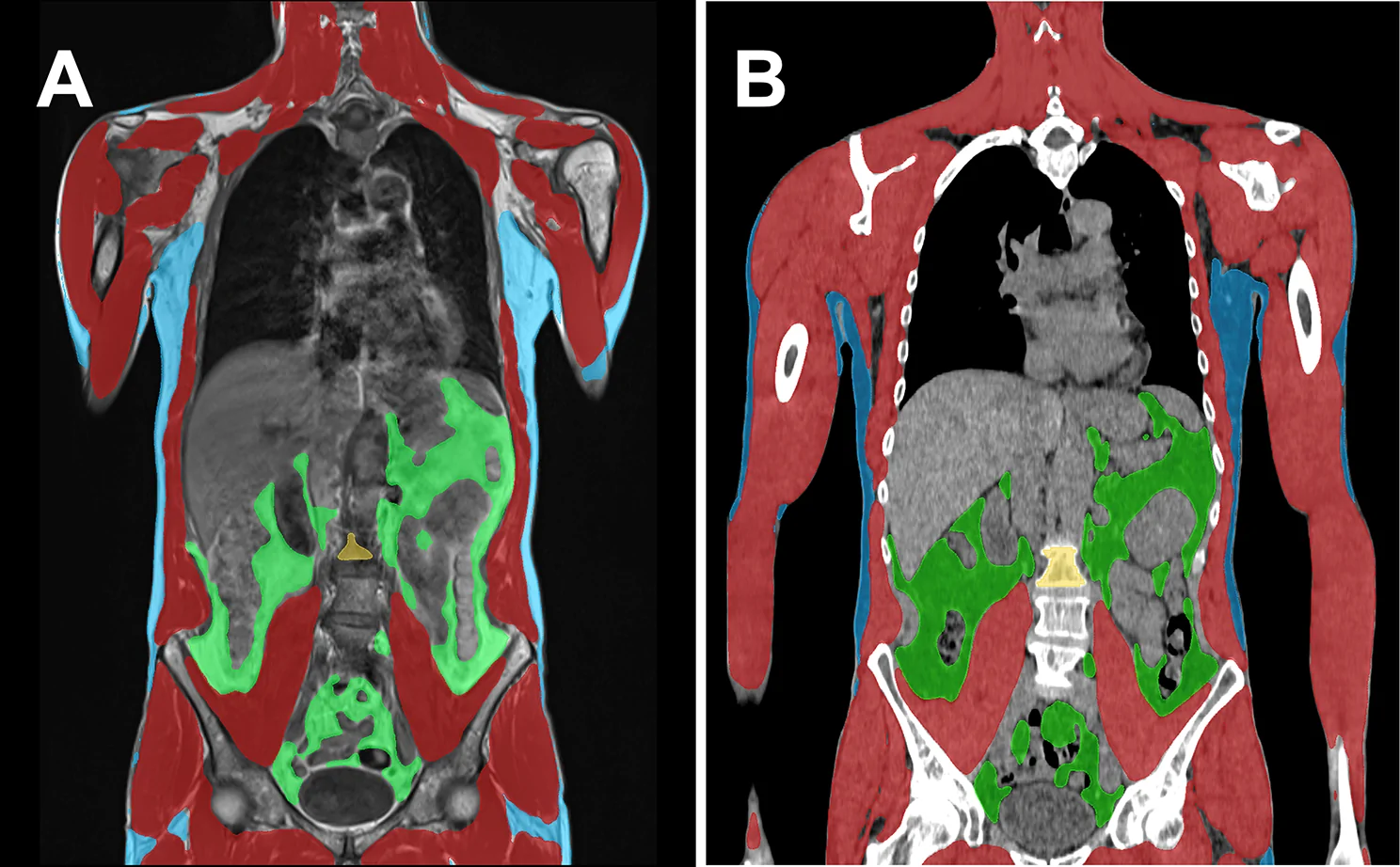
Example images with coronal cutouts of the thoracoabdominal region acquired wit (**A**) Coronal whole-body T1-weighted MRI and (**B**) coronal CT. Color overlays indicate automatically segmented tissues: red = skeletal muscle, blue = subcutaneous adipose tissue, green = visceral adipose tissue

A deep learning-based pipeline was employed, incorporating the TotalSegmentator [7] for CT and TotalSegmentator MRI for MRI [8], respectively, to generate volumetric image segmentations. As a first step, images were anonymized and converted to nifti filetype. Normalization was done in accordance to the TotalSegmentator (Version 2.5) pipeline using a 1.5 mm isotropic resampling followed by nnUNet intensity normaliation based on HU statistics for CT images and z-score normalization for MRI. To ensure comparability and reproducibility across patients with differing scan coverage, quantitative analyses were restricted to regions superior to the tibial plateau in both modalities. The ninth thoracic vertebra was used as the reference point to delineate the boundary between the thoracic and abdominal compartments in order to define the abdominal visceral adipose tissue. Bone landmarks were automatically segmented, and for each segmentation the largest connected component was retained. For the tibia, the tibial plateau (top of the tibia) was used. For the ninth toracal vertebra (T9), the superior endplate of the vertebral body was used to define the superior boundary of the abdominal VAT volume. For the third lumbal vertebra (L3), the centre of the vertebral body (mid-vertebra) along the z-axis was used, in accordance with existing literature [16]. Figure 2 shows an example of automatically obtained segmentations for subcutaneous adipose tissue (SAT), abdominal visceral adipose tissue (VAT), and skeletal muscle (SM), once from MRI and once from CT. All cases underwent manual inspection, and each segmentation was independently evaluated by a board-certified radiologist with more than 6 years of experience, using a 5-point Likert scale [17]. A score of 5 indicated segmentation comparable to manual slice-wise delineation, a score of 4 reflected good segmentation quality, a score of 3 represented cases with noticeable inaccuracies in the overall assessment which were not yet considered critical for the resulting body composition metrics, a score of 2 indicated major problems, and a score of 1 represented very poor segmentation quality.

### Statistical analysis

No formal sample size analysis was performed, but it was intended to create a dataset of at least comparable size to former intermodal body composition analysis papers, which had included between 21 and 50 patients [18, 19, 20]. Analyses were conducted using Python. Wilcoxon signed-rank test was applied to evaluate paired ordinal measures. Two-sided hypothesis testing was performed, with adjustments for multiple comparisons using the Bonferroni correction. Agreement between measurements was assessed with Bland-Altman plots, Pearson r, and intraclass correlation coefficients ICC (two-way mixed-effects model with absolute agreement and single measurement ICC[3, 1]).

## Results

### Study cohort

Table 1 reports descriptive statistics for the patient cohort. 20 (47.6%) of the 42 patients were female. Mean age was 59.9 ± 6.9 years, and mean BMI was 26.4 ± 4.2 kg/m².

**Table 1:** Patient characteristics and body composition metrics descriptive statistics of patient characteristics and body composition variables for the study cohort. Data are presented as mean ± standard deviation (SD)

		Female	Male	Overall
Number of patients		20	22	42
BMI		25.86 ± 4.90	26.90 ± 3.53	26.40 ± 4.21
Age [y]		60.45 ± 7.34	59.32 ± 6.61	59.86 ± 6.91
Size [m]		1.64 ± 0.06	1.78 ± 0.07	1.72 ± 0.10
Weight [kg]		69.30 ± 11.59	85.64 ± 12.47	77.86 ± 14.49
Oncological Diagnosis		All newly diagnosed multiple myeloma	All newly diagnosed multiple myeloma	All newly diagnosed multiple myeloma
CT	Skeletal Muscle [L]	16.04 ± 2.89	25.27 ± 2.79	20.88 ± 5.44
	Subcutaneous Fat [L]	23.29 ± 8.09	16.46 ± 6.31	19.71 ± 7.92
	Visceral Abdominal Fat [L]	2.78 ± 1.46	4.74 ± 2.13	3.81 ± 2.07
MRI	Skeletal Muscle [L]	14.10 ± 2.45	22.10 ± 2.94	18.29 ± 4.85
	Subcutaneous Fat [L]	22.82 ± 7.30	16.38 ± 5.39	19.45 ± 7.09
	Visceral Abdominal Fat [L]	2.58 ± 1.57	4.52 ± 2.25	3.59 ± 2.17

### Agreement and correlation between body composition metric from CT and MRI

**Fig. 3:**
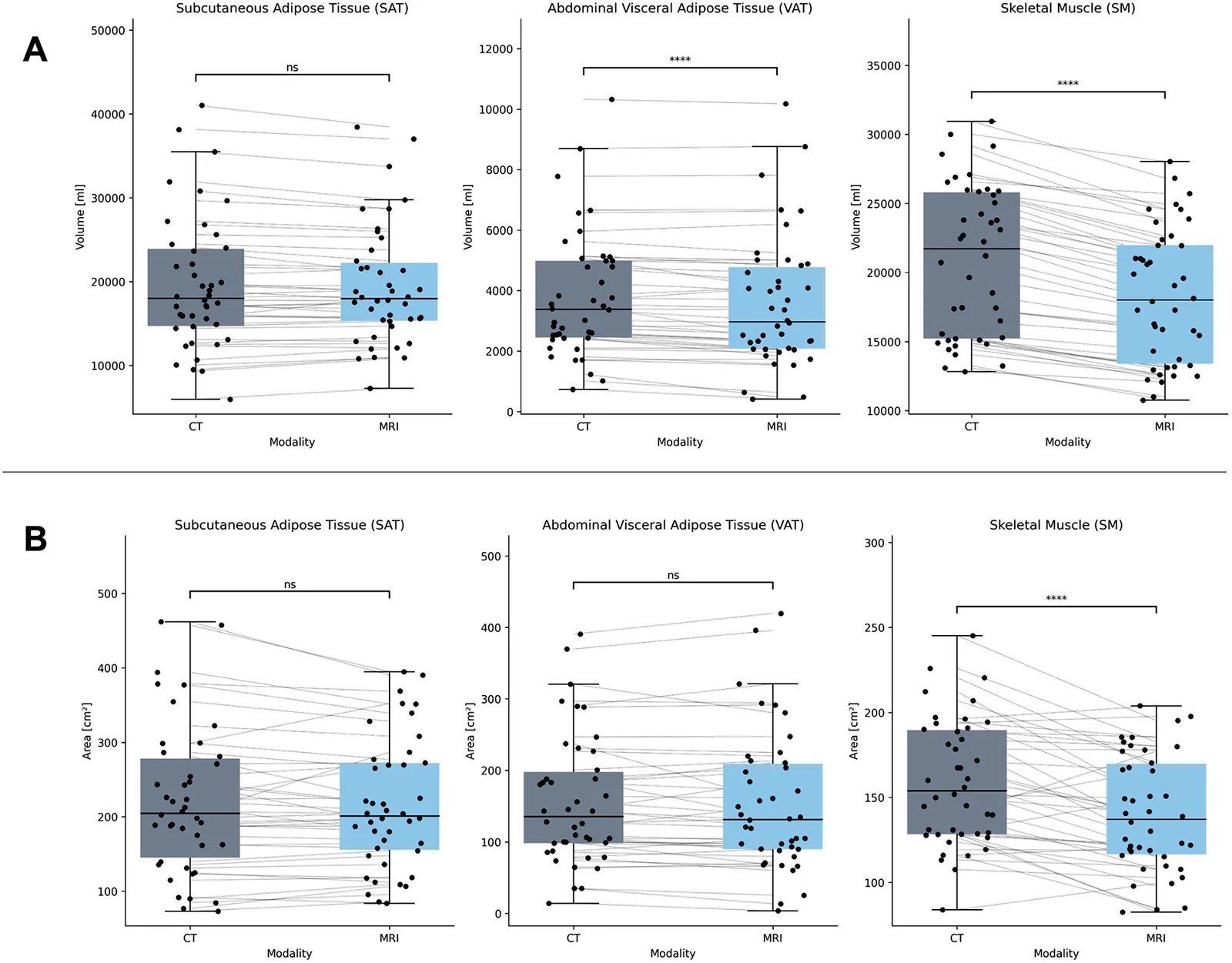
Distribution of body composition measures. Boxplots show subcutaneous adipose tissue (SAT), abdominal visceral adipose tissue (VAT), and skeletal muscle (SM). (**A**) Whole-body volumetric analysis. (**B**) Single axial-slice 2D analysis at the height of third lumbar vertebra (L3). ns = not significant. **** *p* < 0.001

**Fig. 4:**
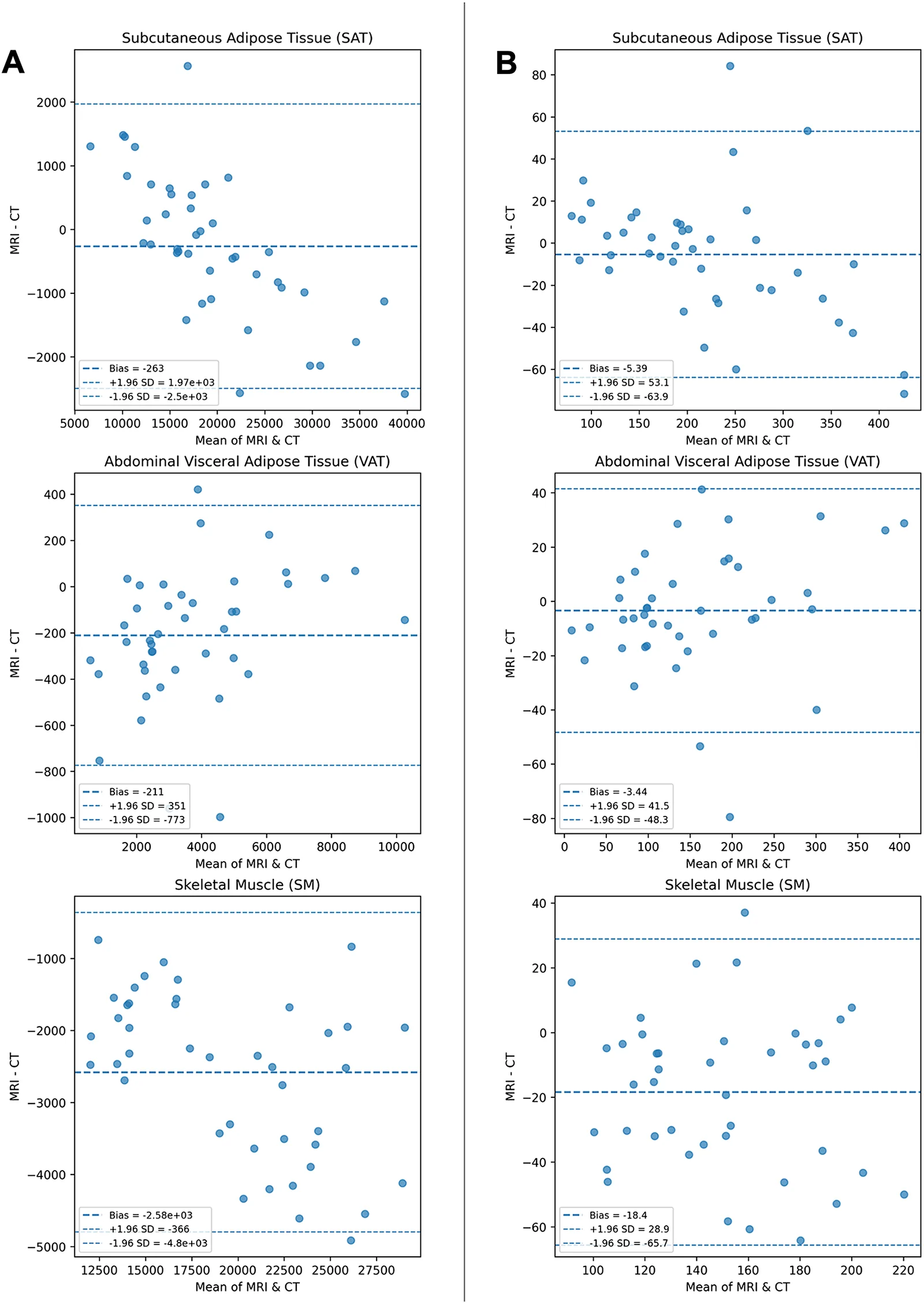
Agreement between CT and MRI. Bland-Altman plots show differences in subcutaneous adipose tissue (SAT), abdominal visceral adipose tissue (VAT), and skeletal muscle (SM) between CT and MRI. (**A**) Whole-body volumetric analysis in ml. (**B**) Single slice 2D analysis in cm^2^. Negative values indicate lower values of MRI compared to CT. In each Bland-Altman plot, differences (MRI-CT) are plotted against the mean of MRI and CT. The central, lightly dashed line indicates the mean difference (bias), and the upper and lower 95% limits of agreement (bias ± 1.96 SD) are shown as more prominently dashed lines

CT and MRI demonstrated high agreement for whole-body volumetric (3D) body composition metrics (Table 2; Figs. 3 and 4). For 3D analysis, concordance was high for SAT (ICC 0.99; 95% CI 0.99-1.00), SM (ICC 0.98; 95% CI 0.98–0.99), and VAT (ICC 0.99; 95% CI 0.99-1.00). Pearson correlations were strong (SAT *r* = 0.99; SM *r* = 0.98; VAT *r* = 0.99; all *p* < 0.0001). Bland-Altman plots demonstrated systematic modality-related differences between MRI and CT (Fig. 4A): both SM (bias: -2582 ml, *p* < 0.0001) and VAT (bias: -211 ml, *p* < 0.0001) were significantly lower when extracted from MRI compared to CT. SAT was not significantly lower when extracted from MRI compared to CT (bias: -263 ml, *p* = 0.1629).

For the single-slice (2D) analysis, agreement remained high for SAT (ICC 0.97; 95% CI 0.95–0.99) and VAT (ICC 0.98; 95% CI 0.97–0.99), while concordance was lower for SM (ICC 0.87; 95% CI 0.76–0.93) (Table 2). Correlations mirrored these results (SAT *r* = 0.95; VAT *r* = 0.97; SM *r* = 0.77; all *p* < 0.0001). Bland-Altman plots showed a systematic modality-related difference (Fig. 4B), with SM being significantly lower when obtained from MRI than from CT (bias: -18.4cm^2^, *p* < 0.0001). SAT (bias: -5.4cm^2^, *p* = 0.25) or VAT (bias: -3.4cm^2^, *p* = 0.35) were also lower when obtained from MRI than from CT, however this was not statistically significant.

**Table 2:** Agreement and correlation of body composition metrics between MRI and CT in both 3D and 2D analyses for subcutaneous adipose tissue (SAT), abdominal visceral adipose tissue (VAT), and skeletal muscle (SM). ICC denotes the intra-class correlation coefficient

	Agreement:ICC [95% CI], p-value	Correlation:Pearson r, p-value
Volumetric 3D analysis		
SAT	0.99[0.99-1.0],1,44E-21	0.99, 5,71E-41
SM	0.98[0.98-0.99],4,75E-15	0.98, 9,71E-31
VAT	0.99[0.99-1.0],1,37E-23	0.99, 2,16E-37
Single-slice 2D analysis		
SAT	0.97[0.95 0.99],1.6470E-22	0.95, 2.1142E-23
SM	0.87[0.76 0.93],8.2166E-10	0.77, 2.0887E-09
VAT	0.98[0.97 0.99],4.3387E-27	0.97, 1.0865E-26

### Segmentation quality assessment and identification of failure modes

To evaluate whether modality-specific differences could be explained by systematic segmentation errors, all segmentations were visually reviewed by a board-certified radiologist. Overall segmentation quality was high, with > 95% of segmentations rated as sufficient (Likert ≥ 3) for both CT and MRI across compartments. Evaluations on a 5-point Likert scale yielded mean scores of 3.8 for abdominal fat (2: 2 (4.8%), 3: 3 (7.1%), 4: 37 (88.1%)), 3.8 for skeletal muscle (2: 2 (4.8%), 3: 3 (7.1%), 4: 37 (88.1%)), and 3.9 for subcutaneous fat (2: 1 (2.4%), 3: 1 (2.4%), 4: 40 (95.2%)) on CT, and on MRI 3.7 for abdominal fat (3: 13 (31.0%), 4: 29 (69.0%)), 3.7 for skeletal muscle (3: 12 (28.6%), 4: 30 (71.4%)), and 3.9 for subcutaneous fat (3: 8 (19.0%), 4: 34 (81.0%)), respectively. These findings indicate consistently good segmentation quality across tissue compartments, with only minor deviations between modalities.

**Fig. 5:**
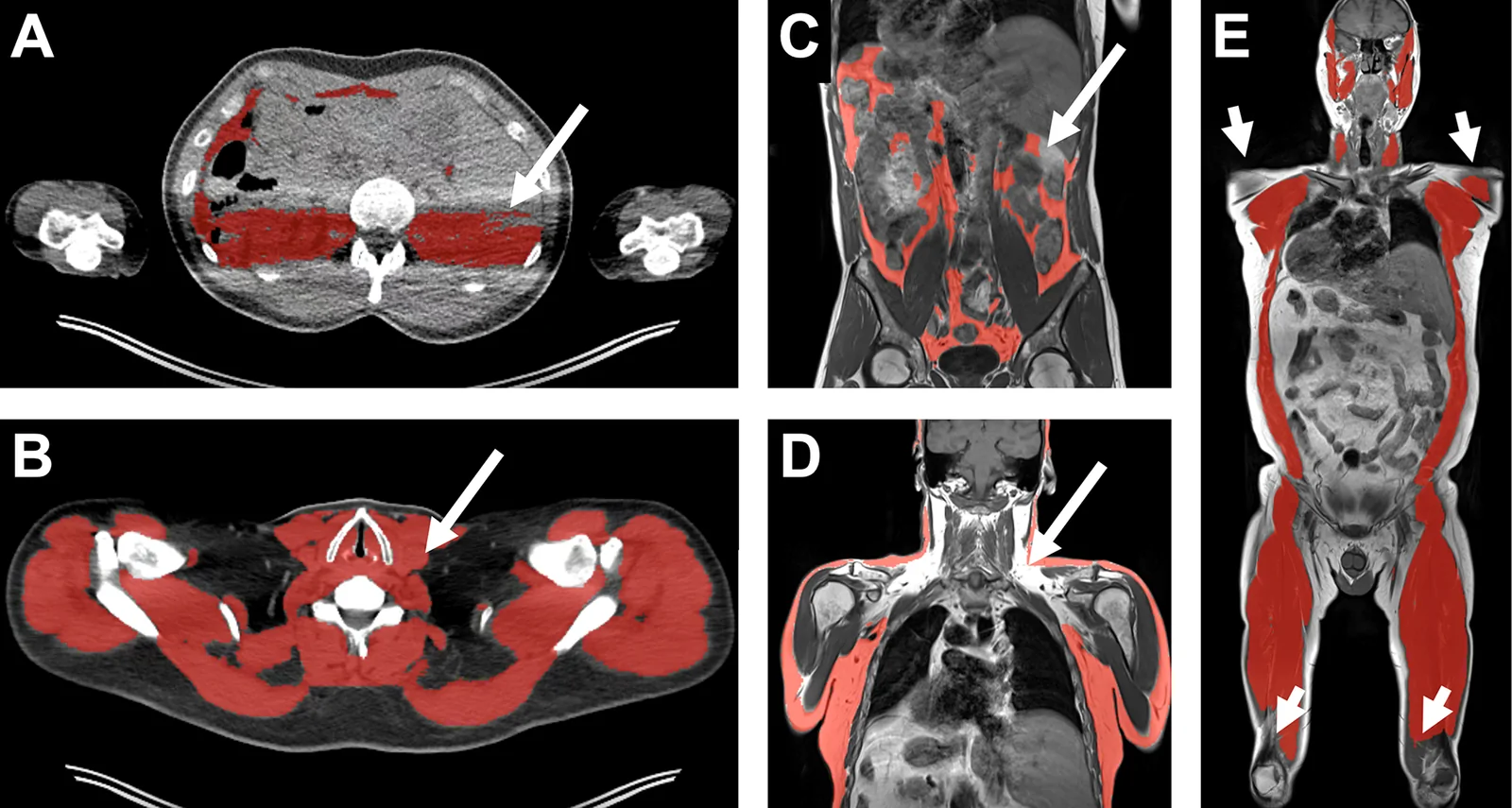
Common failure modes of the segmentation model for CT and MRI. White arrows indicate regions of misclassification. (**A**) Axial CT with artifact leading to incorrect classification of parts of abdominal organs as visceral fat. (**B**) Axial CT of the cervical region with vessels erroneously classified as skeletal muscle. (**C**) Coronal MRI with missing visceral-fat segmentation. (**D**) Coronal MRI with missed subcutaneous fat in the cervical region. (**E**) Coronal MRI showing incomplete muscle segmentation

**Fig. 6:**
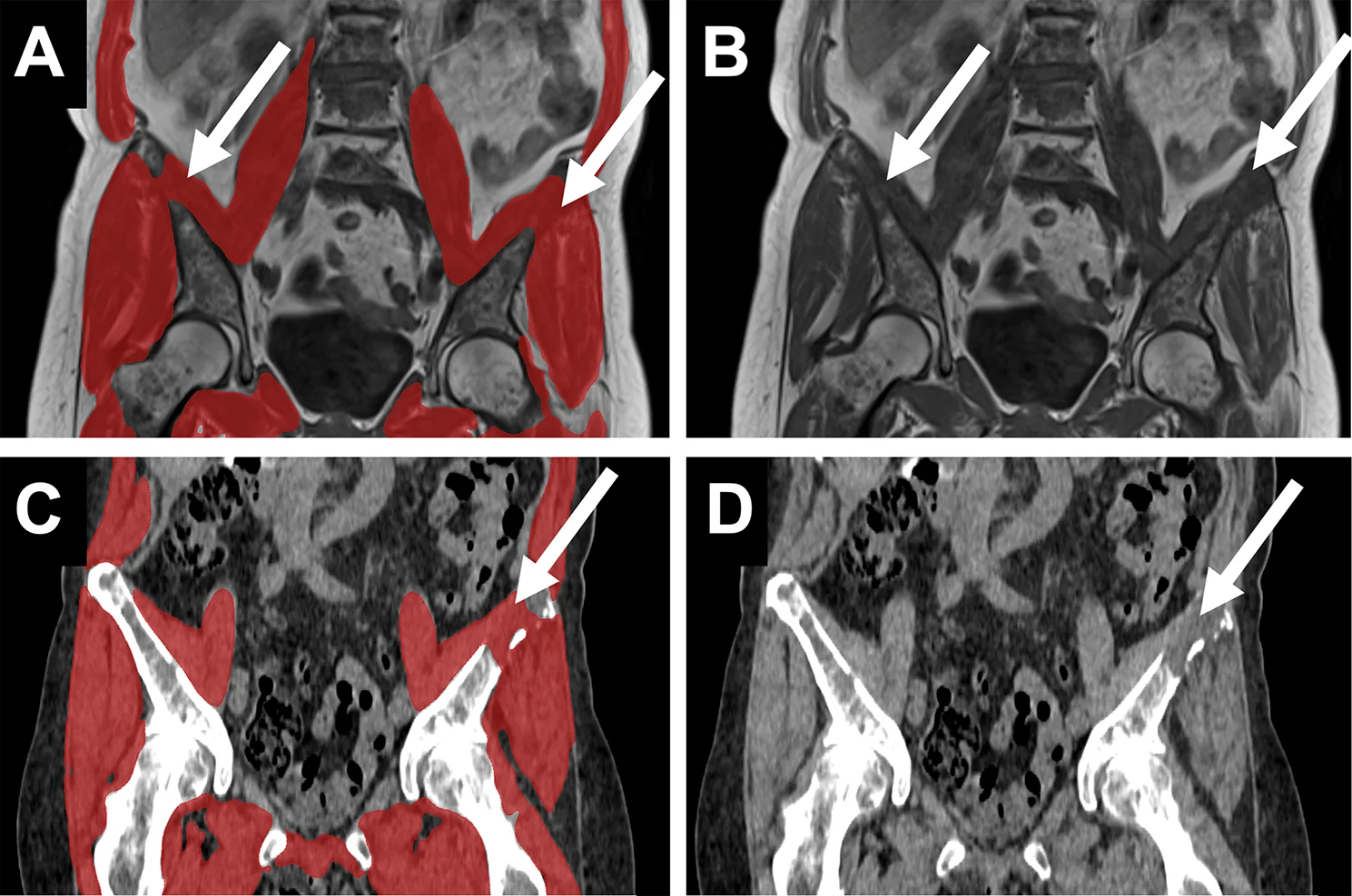
Example of erroneous muscle-tissue classification caused by oncologic pathology in both MRI and CT. The same patient is shown in coronal MRI with overlay for skeletal muscle segmentation (**A**) and without overlay (**B**), and in coronal CT with overlay for skeletal muscle segmentation in (**C**) and without overlay in (**D**). The white arrow marks the region of misclassification. A large focal bone marrow lesion with cortical destruction is located at the left iliac crest and is falsely segmented as skeletal muscle both in CT and in MRI. Additionally, a smaller focal lesion is located on the right iliac crest, which is falsely segmented as skeletal muscle in MRI, but not in CT

Few failure modes which occurred in several cases were identified and primarily related to artifacts or challenging anatomy (Figs. 5 and 6). On CT, errors included artifact-driven misclassification (e.g., abdominal organs labeled as visceral fat) and erroneous labeling of cervical vascular structures as muscle. On MRI, missing VAT segmentation possibly due to bowel motion and partial volume artifacts, and occasional missed segmentation of cervical subcutaneous fat was observed, and incomplete delineation of muscle boundaries occurred. Noteworthy, was also observed that a large myeloma-typical focal bone marrow lesion, which replaced fatty bone marrow with soft tissue and therefore mimics the density and signal intensity of muscle in both CT and MRI, respectively, was misclassified as part of skeletal muscle (Fig. 6).

## Discussion

Imaging-derived body composition metrics are increasingly recognized as prognostic markers [21], yet current evidence mostly relies on CT-derived two-dimensional analyses. With the expanding implementation of MRI and deep learning-based volumetric approaches [10] for body composition assessment, their inter-modality comparability remains insufficiently understood. In this study, we evaluated the intermodal reproducibility of automatically obtained body composition metrics based on same-day CT and MRI examinations. While both volumetric and single-slice body composition measures showed strong correlations and high reproducibility across modalities, we identified systematic modality-specific biases: whole-body T1w-MRI led to significantly lower volumetric VAT and skeletal muscle mass when compared to CT imaging. For slice-wise analysis at the L3 level, significantly lower values for skeletal muscle were observed for MRI compared to CT.

For single-slice analysis, our results are broadly consistent with previously reported findings on CT-MRI body composition comparisons. For 2D assessments, Faron et al. [18] found ICC > 0.99 for skeletal muscle with CT averaging + 3.6 cm² versus MRI on a single SMA-level slice (psoas/quadratus lumborum excluded; manual segmentations). Klopfstein et al. [19] reported a Pearson *r* ≈ 0.9, with mean biases of -2.9% for VAT and + 0.4% for SAT comparing MRI and CT at the level of the umbilicus, thus showing slightly lower measurements for MRI than for CT. Hush et al. [20] found Pearson *r* > 0.97 and mean biases up to -2 cm² for SAT and VAT on lumbar slices, yielding slightly lower values for MRI than for CT.

Although 3D volumetric comparisons have been reported for epi- and pericardial adipose tissue [22, 23], to our knowledge no direct MRI-CT comparisons have been published for whole-body volumetric analysis. Therefore, our study represents, to the best of our knowledge, the first direct MRI-CT comparison for AI-based body composition at the whole-body level.

Several factors may explain the observed differences between volumetric whole-body MRI and CT measurements. First, visual inspection and quality control revealed minor segmentation inaccuracies at anatomical borders, despite overall satisfactory segmentation quality. These discrepancies are likely attributable to partial volume effects and motion artifacts due to MRI signal characteristics that pose not only an inherently greater challenge to humans but also to deep learning models. Second, in some cases, the model appeared to misclassify parts of the cervical region such as vascular structures, as muscle tissue in the CT images, potentially influenced by the non-contrast imaging protocol or an underrepresentation in the training data. Third, because our analysis included the extremities, variability may have arisen from regional differences in scan coverage, with the upper extremities sometimes not fully within the field of view and the lower extremities often truncated at different locations of the tibia. To ensure consistency, all scans were analyzed from head to tibia, providing a standardized basis for comparison in the lower extremities. Nonetheless, variations regarding inclusion of the upper extremities in the respective images, which are sometimes only partially captured within the field of view in whole-body MRI, may have introduced additional discrepancies.

This study cohort consists of patients with newly diagnosed multiple myeloma, a population in which body composition has been associated with key clinical outcomes [20, 32], including overall survival. However, existing multiple myeloma studies show substantial heterogeneity in both methodology and reported associations. Most prior analyses have relied on two dimensional measures (23–31], with only few incorporating volumetric approaches [33], and are mostly limited to the use of PET/CT [34]. As MRI more and more assumes a central role in the diagnosis and treatment monitoring in multiple myeloma [35, 36], our findings indicate that AI-based body composition analysis from whole-body MRI would yield comparable results to those known from PET/CT literature, and that correction for the observed modality specific biases might improve comparability between PET/CT and MRI based body composition metrics.

Several limitations have to be noted. Although the whole-body MRI protocol used in this analysis is widely used for multiple myeloma imaging in our multicentric trials, MRI imaging protocols vary across different oncological entities, and our results may only partially generalize to other MRI sequences or acquisition settings. Furthermore, results are limited to the TotalSegmentator-based automated deep learning segmentation models [7, 8] and performance of other customized segmentation models may vary depending on training data and annotation protocols [37]. Future studies should validate findings across diverse MRI protocols, reconstruction kernels, and deep learning models, and assess clinical integration in prospective trials.

## Conclusion

While intermodal agreement for AI-based body composition metrics between (PET-) CT and whole-body MRI was generally high, especially for volumetric composition metrics specific intermodal biases exist. These biases should be considered when pooling multimodal imaging data for research analysis, when comparing results from multiple studies in case these were obtained with different imaging modalities, and for prospective integration of body composition analysis in large-scale clinical practice.

## Supplementary Information

Below is the link to the electronic supplementary material.

Supplementary Material 1

Supplementary Material 2

Supplementary Material 3

## Data Availability

The patient level imaging and clinical data used for this study is not publicly available, because it contains protected patient health information and is subject to EU data protection laws. Code is available on request.
