# Supplementary material for: AI-based automated body composition analysis from whole-body MRI and (PET-) CT: an intermodal comparison from same-day examinations
Source: Cancer Imaging. 2026 Oct 24;26:149. doi: 10.1186/s40644-026-01134-5 (PMC13700498; doi:10.1186/s40644-026-01134-5)
Supplement: Supplementary file 1 [file 40644_2026_1134_MOESM3_ESM.pdf]

Supplementary Material

Imaging Protocol:

Whole-body MRI was performed on a 1.5 T system (Magnetom Aera; Siemens Healthineers, Erlangen, Germany; software version syngo MR E11) equipped with the XQ gradient engine (maximum gradient amplitude 45 mT/m and maximum slew rate 200 mT/m/s per axis). Signal was received using a combination of Head/Neck 20, two Body 18, and Peripheral Angio Feet 36 coils for the whole-body protocol, and a single Body 18 coil for the dedicated pelvic acquisition. The protocol comprised a coronal T1-weighted turbo spin-echo (TSE) sequence, a coronal T2-weighted short-tau inversion recovery (STIR) TSE sequence, and axial diffusion-weighted echo-planar imaging (DWI). Automated body composition analysis was based on the coronal T1-weighted TSE sequence (TR/TE 528/8.4 ms, flip angle 150°, one signal average; slice thickness 5.0 mm with a 10% distance factor; field of view 375 × 500 mm; acquisition matrix 432 × 768; interpolated in-plane resolution 1.3 × 1.3 mm; GRAPPA acceleration factor 2), acquired over five whole-body stations with 375 mm z-axis coverage and an acquisition time of 1 min 43 s per station.

^{18}F-FDG PET/CT scans were performed on the Biograph System (Siemens Healthineers, Erlangen, Germany), specifically a Biograph 128 Vision Quadra Edge (n=22, 52%), Biograph mCT 40 (n=14, 33%) and Biograph mCT 20 (n=6, 14%). ^{18}F-FDG PET/CT scans were performed 60 minutes after intravenous administration of ^{18}F-FDG, covering the region from the skull to the toes, using a Biograph mCT (Siemens Healthineers, Erlangen, Germany). Low-dose CT scans (120 kV, 30 effective mAs) were acquired for both attenuation correction and anatomical co-registration. The CT component was used for the CT-based body composition analysis.
